# Extramural vascular invasion detected by contrast-enhanced multiple-row detectors computed tomography (ceMDCT) as a predictor of synchronous metastases in colon cancer

**DOI:** 10.18632/oncotarget.22034

**Published:** 2017-10-25

**Authors:** Su-Xing Yang, Xun Yao, Xing-He Song, Yan-Cheng Cui, Ying-Jiang Ye, Yi Wang

**Affiliations:** ^1^ Department of Radiology, Aerospace Center Hospital, Beijing 100049, China; ^2^ Department of Gastrointestinal Surgery, Peking University People’s Hospital, Beijing 100044, China

**Keywords:** colon cancer, synchronous metastases, contrast-enhanced computed tomography, extramural vascular invasion

## Abstract

**Background:**

Extramural Vascular Invasion (EMVI) is histologically defined as the presence of tumor cells beyond the muscularis propria in vessels resulting in disease metastases.

**Objective:**

To determine whether EMVI, detected by contrast-enhanced multiple-row detectors computed tomography (MDCT), has closely association with synchronous metastases in colon cancer.

**Methods:**

Patients with pathology proven colon cancer were included in this retrospective study. Preoperative imaging status, including Extramural tumor depth, Lymph nodes, tumor location, and ctEMVI status, were defined on MDCT. Postoperative pathological tumor stage, lymph node stage, and tumor differentiation, were defined in accordance with the American Joint Committee on Cancer (AJCC) 7^th^ Edition. Synchronous metastases were detected on follow-up MDCT 3 months after initial diagnosis or by surgery, if available. Associations between ctEMVI and other preoperative and postoperative factors were analyzed using Chi-squared tests. Logistic regression analyses were performed to analyze the preoperative and postoperative factors of synchronous metastases in colon cancer.

**Results:**

ctEMVI was observed in 96 patients (96/241, 39.8%). The presence of ctEMVI varied significantly depending on ctEMD (χ2 = 66.557, *P*<0.001), lymph nodes status on MDCT (χ2 =24.533, *P=*0.001), pathological tumor status (χ2 = 36.267, *P* <0.001) and pathological lymph nodes status analyses (χ2 =32.103, *P* <0.001). Synchronous metastases were seen in 36 patients (36/96, 37.5%) with ctEMVI and 11 (11/145, 7.6%) patients without ctEMVI. The incidence of synchronous metastases was significantly higher in the cohort of positive ctEMVI with odds ratio (OR) of 7.309 (95% CI 3.485∼15.330, *P*<0.001). Positive ctEMVI (Odds ratio 4.654, 95%CI: 1.987∼10.898, *P* <0.001) and ctEMD larger than 5 mm (Odds ratio 2.654, 95%CI: 1.116∼6.309, *P* =0.027) were demonstrated to be significant preoperative factors in predicting synchronous metastases.

**Conclusion:**

MDCT-detected EMVI could be used as a preoperative factor to predict synchronous metastases in colon cancer.

## INTRODUCTION

Colorectal cancer has become the third most common cancer worldwide and the fourth leading cause of death from cancer [1]. Synchronous distant metastases were detected in 14.5% of colorectal cancer patients when initial diagnosis was made [2]. Surveillance of synchronous distant metastases in patients with locally advanced colon cancer is very important for management of treatment.

Contrast-enhanced thoracic abdominal and pelvic MDCT had been used as the clinical routine methods to detect metastases preoperatively. However, the accuracy of MDCT limited by poor soft-tissue resolution and scanning range [3]. MRI can be considered as a suitable imaging modality for determining potentially resectable hepatic metastases suggested by the 2017 National Comprehensive Cancer Network (NCCN) guidelines. A recent meta-analysis had found that the sensitivity of Magnetic Resonance Imaging (MRI) was reported as 91.0-97.0% in identification of liver metastases [4, 5], especially for metastases equal to or smaller than 1 cm in diameter [6]. However, it is not yet clear that liver MRI should be performed as a first-line examination in patients with colorectal cancer in addition to standard staging MDCT at baseline and each follow-up scan. Han K etc. found that staging liver MRI is likely unnecessary for patients without suspicious hepatic findings on MDCT, although it can be performed to further confirm benignity of small hepatic lesions [7]. Furthermore, many studies had confirmed that FDG PET/CT is not yet clear for the small hepatic lesions, whose diameter of the lesions was smaller than 1 centimeter [5]. Based on these, selecting patients with high risk preoperatively to receive further MRI or PET/CT scan is important in precision treatment and saving medical resources.

It is known that tumor status and lymph nodes status are closely associated with synchronous metastases. But, the incidence of synchronous metastases was different for the patients with same pathological status [8, 9]. EMVI was histologically defined as the presence of tumor cells beyond the muscularis propria in vessels [10, 11]. Many studies have confirmed that EMVI is an adverse prognostic factor for survival in patients with colorectal cancer, especially in those without lymph node metastases [10, 12, 13]. Sohn, et al. found that MRI-detected extramural vascular invasion (EMVI) is an independent high-risk factor for synchronous metastases in patients with rectal cancer [14]. Most of this literature concentrated on EMVI detected by MRI or pathology. However, few studies have connected MDCT-detected EMVI with synchronous metastases. On the basis of previous studies cited above, we hypothesize that MDCT-detected EMVI could associated with synchronous metastases. The objective of this study is to assess whether MDCT-detected EMVI could significantly associated synchronous metastases in patients with colon cancer.

## RESULTS

### Patients

**Figure 1:**
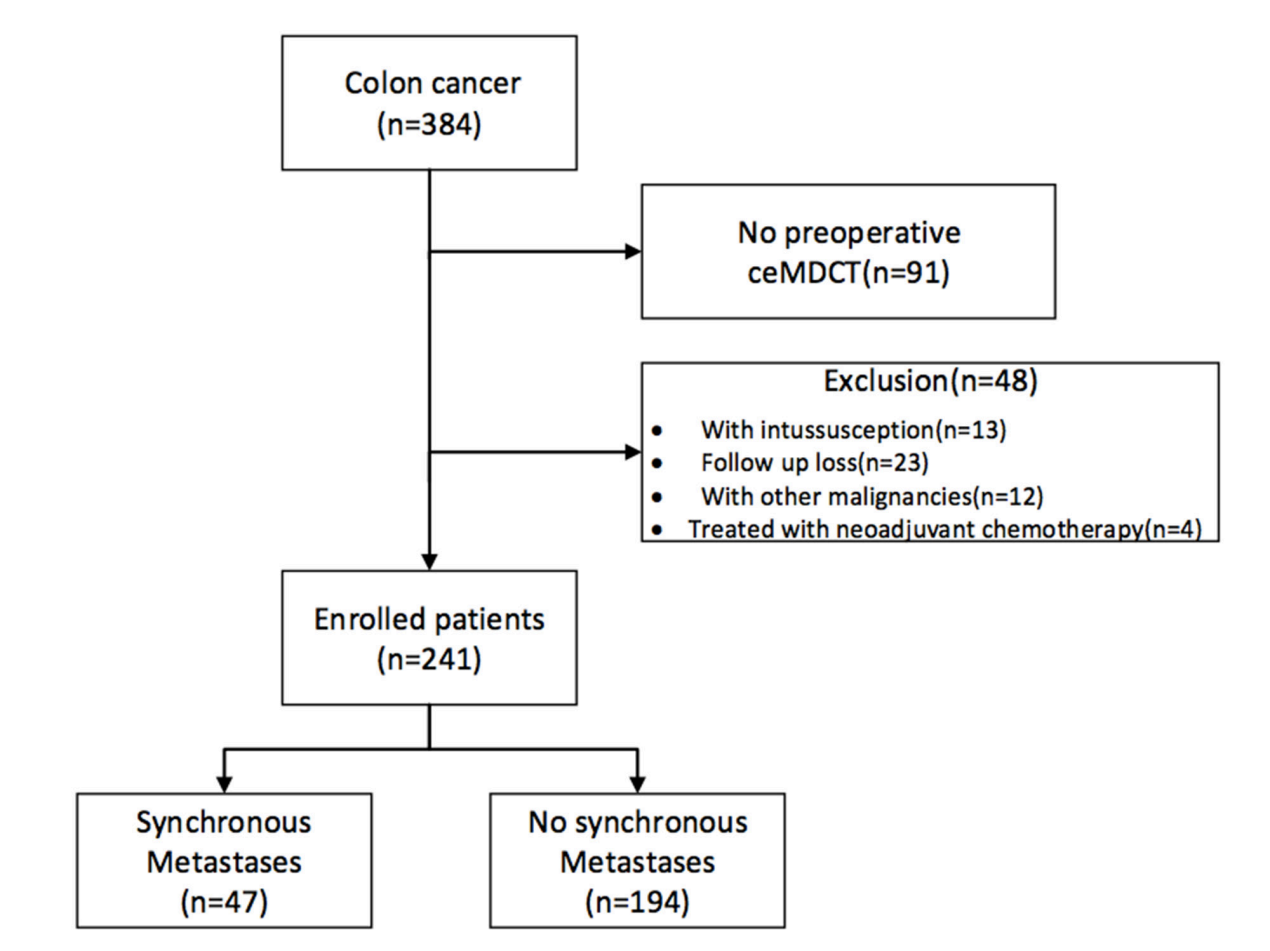
Flowchart for patient recruiting in this study

Three hundred and eighty-four patients with biopsy-proven colon cancer underwent curative surgery from January 2009 to December 2013. One hundred and forty-three patients were excluded based on the criteria: 91 patients did not undergo preoperative enhanced MDCT images in our hospital; 13 patients who had complicated intussusception underwent emergency surgery; 12 patients were detected with combined malignant tumor; 4 patients were treated with neoadjuvant chemotherapy; 23 patients were lost to follow-up examination at least 3 months after initial surgery. Finally, 241 colon cancer patients were included in this study (Figure 1). There were 122 males (50.6%) and 119 females (49.4%) patients. The median age of the patients was 70 years with an interquartile range of 59∼77 years. All patients underwent curative surgery within 3 weeks after an initial diagnosis was made.

### Incidence of synchronous metastases

Synchronous metastases were confirmed in 47 patients (47/241, 19.5%). Only 6 patients underwent PET/CT and/or superior abdomen MR for further scan after MDCT. Eighteen patients had confirmed metastases by pathological analyses after surgery.

Synchronous metastases included hepatic metastases in 22 patients (22/47, 46.8%), peritoneal metastases in 8 patients (8/47, 17.0%) and lung metastases in 5 patients (5/47, 10.6%). Nine patients (9/47, 19.1%) had synchronous metastases that involved two organs. Two patients (2/47, 4.2%) had liver, lung and peritoneal metastases. One patient (1/47, 2.1%) had bone metastases.

### The association between the ctEMVI and the parameters

Good agreements were obtained between the two observers regarding the identification of ctEMVI and ctEMD categories with k values of 0.702 and 0.664, respectively. And moderate agreement of ctN with the k value of 0.487.

ctEMVI was detected on MDCT in 96 patients (96/241, 39.8%). There was a statistically significant relationship between ctEMD, ctN, pT and pN. And there was no statistically significant relationship between ctEMVI and the covariates including age, sex, tumor location, and tumor differentiation (Table 1).

**Table 1:** Association between presence of extramural venous invasion detected on MDCT (ctEMVI) and preoperative and postoperative factors

		Total (n=241) N (%)	EMVI present (n=96) N (%)	EMVI absent (n=145) N (%)	χ^2^	*P* value
Age	<65	95(39.4)	42(43.8)	53(36.6)	1.253	0.283
	≥65	146(60.6)	54(56.3)	92(63.4)		
Sex	male	122(50.6)	50(52.1)	72(49.7)	0.136	0.793
	female	119(49.4)	46(47.9)	73(50.3)		
**Preoperative parameters**						
ctN	Negative	91(17.8)	18(18.8)	73(50.3)	24.533	<0.001
	Positive	150(62.2)	78(81.3)	72(49.7)		
ctEMD	<5mm	135 (56.0)	23(24.0)	112(77.2)	66.557	<0.001
	≥5mm	106(44.0)	73(76.0)	33(22.8)		
Location	right	132(54.8)	52(54.2)	80(55.2)	0.024	0.895
	left	109(45.2)	44(45.8)	65(44.8)		
**Postoperative parameters**						
pT	≤T2	25(10.4)	1(1.0)	24(16.6)	36.267	<0.001
	T3	35(14.5)	5(5.2)	30(20.7)		
	T4a	142(58.9)	64(66.7)	78(53.8)		
	T4b	39(16.2)	26(27.1)	13(9.0)		
pN	N0	107(44.4)	26(27.1)	81(55.9)	32.103	<0.001
	N1	71(29.5)	27(28.1)	44(30.3)		
	N2	63(26.1)	43(44.8)	20(13.8)		
Differentiation	High-Medium	164(68.0)	59(61.5)	105(72.4)	3.188	0.090
	Low	77(32.0)	37(38.5)	40(27.6)		

^1^ ctEMVI: CT detected extramural vascular invasion; ctN: CT detected lymph node status; ctEMD: CT detected extramural tumor depth; pT: pathological tumor status; pN: pathological lymph node status.

### Parameters of 182 patients with synchronous metastases

**Figure 2:**
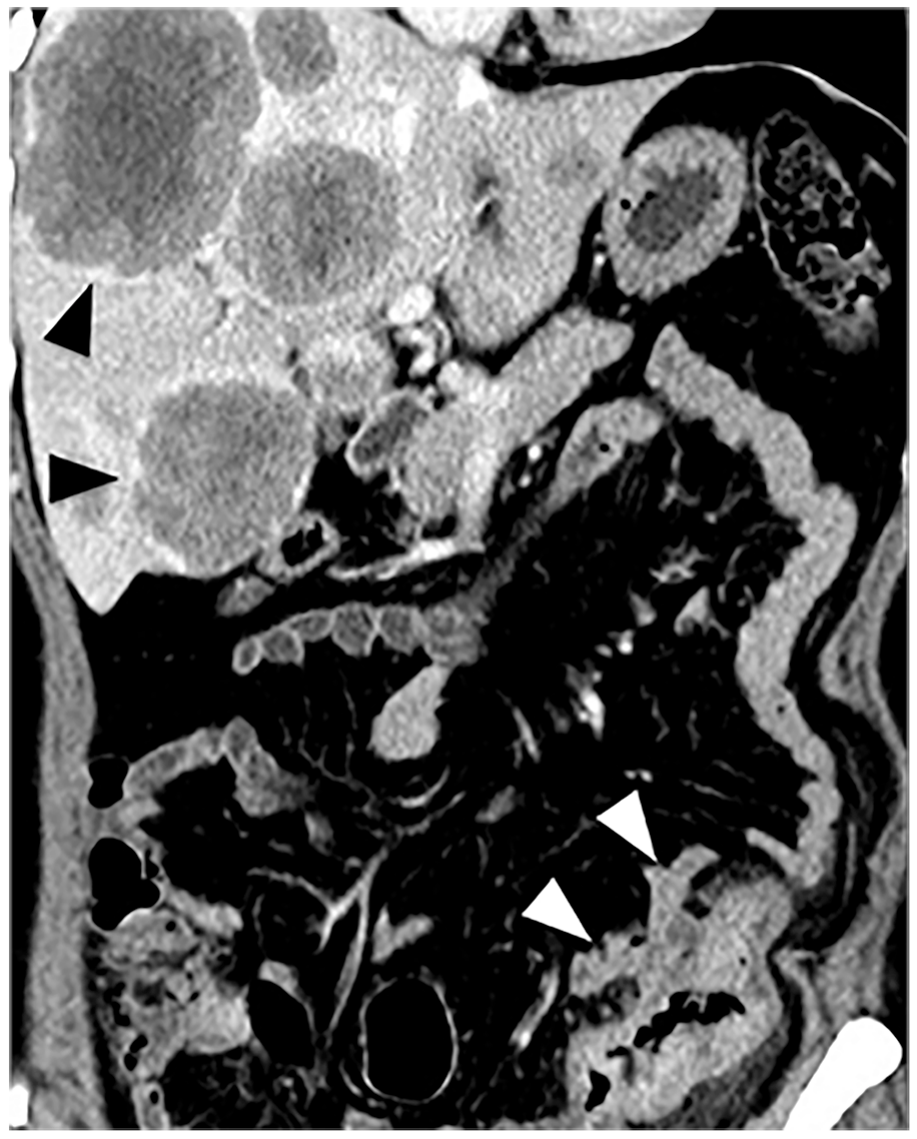
Preoperative MDCT image shows sigmoid cancer of a 79-year-old female Tumor demonstrates evidence of EMVI (white arrow) with liver metastases.

Synchronous metastases were obtained in 36 patients with positive ctEMVI (36/92, 37.5%) and in 11 patients (11/145, 7.6%) with negative ctEMVI. The incidence of synchronous metastases was significantly higher in the cohort of positive ctEMVI with odds ratio (OR) of 7.309 (95% CI 3.485∼15.330, *P*<0.001) (Figure 2).

In the group of patients with metastases, preoperative and postoperative factors were considered to indicate significant difference at the 0.05 levels. Finally, multivariable logistic regression found that positive ctEMVI (odds ratio (OR) = 4.654, 95%CI: 1.987∼10.898, *P* <0.001) and ctEMD larger than 5 mm (OR=2.654, 95%CI: 1.116∼6.309, *P* =0.027) were demonstrated as high-risk preoperative factors for synchronous metastases in colon cancer (Table 2). And positive pN were demonstrated as high-risk postoperative factors for synchronous metastases (Table 3).

**Table 2:** Association analysis of synchronous metastases and preoperative factors in colon cancer

		Total (n=241) N (%)	SM (−) (n=194) N (%)	SM (+) (n=47) N (%)	Univariate analysis		Multivariate analysis			
					χ^2^	*P* value	*P* value	Odds ratio	95% CI	
									Lower	Higher
Age	<65	95(39.4)	80(41.2)	15(31.9)	1.377	0.318		Ref		
	≥65	146(60.6)	114(58.8)	32(68.1)			0.061	2.609	0.968	4.422
Sex	male	122(50.6)	101(52.1)	21(44.7)	0.825	0.417		Ref		
	female	119(49.4)	93(47.9)	26(55.3)			0.419	1.343	0.656	2.750
ctEMVI	Negative	145(60.2)	134(69.1)	11(23.4)	32.923	<0.001		Ref		
	Positive	96(39.8)	60(30.9)	36(76.6)			<0.001	4.654	1.987	10.898
ctEMD	<5mm	135(56.0)	124(63.9)	11(23.4)	25.204	<0.001		Ref		
	≥5mm	106(44.0)	70(36.1)	36(76.6)			0.027	2.654	1.116	6.309
ctN	Negative	91(37.8)	82(42.3)	9(19.1)	8.605	0.003		Ref		
	Positive	150(62.2)	112(57.7)	38(80.9)			0.382	1.508	0.600	3.785
Location	right	132(54.8)	103(53.1)	29(61.7)	1.132	0.329		Ref		
	left	109(5.2)	91(46.9)	18(38.3)			0.384	0.719	0.341	1.513

^1^ ctEMVI: CT detected extramural vascular invasion; ctN: CT detected lymph node status; ctEMD: CT detected extramural tumor depth.

**Table 3:** Association analysis of synchronous metastases and postoperative factors in colon cancer

		Total (n=241) N (%)	SM (−) (n=194) N (%)	SM (+) (n=47) N (%)	Univariate analysis		Multivariate analysis			
					χ^2^	*P* value	*P* value	Odds ratio	95% CI	
									Lower	Higher
Age	<65	95(39.4)	80(41.2)	15(31.9)	1.377	0.318		Ref		
	≥65	146(60.6)	114(58.8)	32(68.1)			0.083	2.045	0.911	4.595
Sex	male	122(50.6)	101(52.1)	21(44.7)	0.825	0.417		Ref		
	female	119(49.4)	93(47.9)	26(55.3)			0.523	1.277	0.603	2.708
pT	≤T2	25(10.4)	25(12.9)	0(0)	19.358	<0.001	0.997	0.00	0.00	0.00
	T3	35(14.5)	35(18.0)	0(0)			0.996	0.00	0.00	0.00
	T4a	142(58.9)	105(54.1)	37(78.7)				Ref		
	T4b	39(16.2)	29(14.9)	10(21.3)			0.770	0.876	0.360	2.129
pN	N0	107(44.4)	103(53.1)	4(8.5)	39.243	<0.001		Ref		
	N1	71(29.5)	55(28.4)	16(34.0)			0.008	4.866	1.498	15.803
	N2	63(26.1)	36(18.6)	27(57.4)			<0.001	15.135	4.749	48.229
Differentiation	High-Medium	164(68.0)	138(71.1)	26(55.3)	4.352	0.054		Ref		
	Low	77(32.0)	56(28.9)	21(44.7)			0.162	1.314	0.897	1.926

^1^ ctEMVI: CT detected extramural vascular invasion; ctN: CT detected lymph node status; ctEMD: CT detected extramural tumor depth.

## DISCUSSION

The present study demonstrated that MDCT-detected EMVI and MDCT-detected EMD larger than 5mm could be used as preoperative radiological factors to predict synchronous metastases in colon cancer.

The lymph or blood vessels invaded by gastrointestinal cancer were considered as a crucial step in the process of distant metastases. Compared with intramural vascular invasion, EMVI showed significant association with the occurrence of distant metastases and long-term prognostic impact in pathological study [10, 12, 15]. EMVI on transected images was identified as macroscopic tumor thrombosis in larger peritumoral veins, which was demonstrated as a high-risk factor to predict increased prognostic significance in patients with colorectal cancer [16, 17]. Given the accuracy of advanced high-resolution techniques, MRI was used to assess the detail of local characteristics of rectal cancer including EMVI before operation [11]. Hunter et al. demonstrated that incidence of confirmed distant metastases was significantly greater in the rectal cancer group with positive EMVI (28/135, 20.7%) compared to the group with negative EMVI (4/95, 4.2%), with odds ratio of 6.0 (95% CI 2.0–17.6) [18].

However, it would be difficult to acquire high-resolution MRI for assessing the local characteristics of colon cancer in detail due to the limitation in techniques. MDCT is currently used as the standard modality for evaluations of colon cancer given the advantage of the short scan time and the convenient three-dimensional reconstruction. Dighe et al. reported the accuracy of MDCT in detection of EMVI in patients with colon cancer is 70 % (95%CI: 66 ∼ 84%). [16]. To our knowledge, there are no previous studies that investigated the association between ctEMVI with synchronous metastases in colon cancer. In the present study, the association between synchronous metastases and preoperative and postoperative factors were analyzed. As the results shown, EMVI status detected on MDCT was demonstrated to be a more significant factor, with OR of 4.654 associated with distant metastases. A significantly different rate of synchronous metastases was obtained between EMVI-positive and EMVI-negative within this cohort of colon cancer patients.

A recent meta-analysis showed that the sensitivity, specificity and diagnostic ratio of CT detected EMD <5mm and ≥5mm were 77% (95% CI: 66-85%), 70% (95% CI: 53 to 83%) and 7.8 (95% CI: 4.2 to 14.2) [19]. In order to improve the accuracy of tumor invasion detected by CT, we used ctEMD to describe the tumor invasion. In the present study, the depth of tumor infiltration measured on MDCT larger than 5mm preoperatively demonstrated as high-risk factors associated with synchronous metastases by multivariate analysis. In consistent, previous studies showed that depth of tumor invasion of colon cancer has close association with potential distant metastases [20-22]. Furthermore, serosal penetration was demonstrated by multivariate analysis as a separate pathologic variable that has independent adverse prognostic significance by a number of large studies [21, 23]. While in our study, three quarters of patients were proven pathological T4 stage, and all of the synchronous metastases were occurred in patients with pathological T4 status. Therefore, the association between serosal penetration and synchronous metastases was not demonstrated.

In this study, postoperative pathology proven lymph nodes metastases has closely association with synchronous metastases in colon cancer, which was consistent with previous studies [21, 24, 25]. In comparison, preoperative lymph node status defined on MDCT can’t be used as an imaging biomarker to predict metastases before operation. It may be induced to the lower accuracy of MDCT-detected N stage varies in different studies [19, 26].

We also found that the presence of CT detected EMVI varied significantly depending on preoperative radiological and postoperative pathological tumor and lymph nodes status. David et al. had found that in 1928 patients with colorectal cancer, pathological EMVI is more common in higher tumor and lymph node stage [27]. ctEMVI varied significantly depending on ctEMD in our study, which was similar to the result of pathological tumor and lymph status. Some studies have shown that high-resolution MR diagnosis of EMVI positive can be used as a predictor of lymph node metastasis in colorectal cancer, suggesting that mrEMVI has a significant correlation with lymph node metastasis [28-30]. ctEMVI was associated with ctN in this study, similar to reported in the literature.

Despite its promising potential, further studies are needed to address some of the limitations of this current study. First, EMVI was not analyzed by pathological analyses in the medical records due to the retrospective study. Therefore, the accuracy of ctEMVI could not be validated with histopathological analyses as reference. Second, PET/CT and MRI with liver-specific contrast were not used as routine clinical imaging tools for surveillance of synchronous metastases. Therefore, potential metastases may have been misdiagnosed. Third, EMVI and liver metastases may be presented in the same patient and same picture, which may induce the bias related with detection of EMVI on MDCT. However, we asked for two radiologists to review and reach consensus on EMVI in order to decrease the misdiagnosis. A future study is needed where radiologists prospectively review images and define EMVI status, tumor and lymph node status.

In conclusion, EMVI status detected on MDCT preoperatively, as high-risk factors, were significantly associated with synchronous metastases in colon cancer. Before operation, patients with positive EMVI on MDCT may need a more aggressive imaging strategy utilizing FDG-PET/CT or MRI to screen potential synchronous metastases.

## MATERIALS AND METHODS

### Patients

Our Institutional Review Board approved this retrospective case-control study and waived the requirement for informed consent. Patients were selected from a single hospital’s electronic colon cancer registry. Patients who underwent preoperative contrast-enhanced abdominal and/or pelvic MDCT at our hospital between January 2009 and December 2013 were retrospectively reviewed. In this retrospective study, all included patients underwent curative surgery and had pathology proven colon cancer. Patients lost to follow-up at least 3 months after initial surgery, and/or with other malignancies, and/or with intussusception and/or treated with neoadjuvant were excluded.

In the diagnostic colon cancer workup, all recruited patients underwent abdominal/pelvic MDCT and chest MDCT to scan for distant metastases. To build a standard reference for synchronous metastases, the electronic medical records of enrolled patients were reviewed retrospectively.

### MDCT technique and image acquisition [31]

MDCT exams were performed on a Siemens SOMATOM Sensation 64 MDCT (Siemens Healthineers, Erlangen, Germany), a GE LightSpeed VCT 64-row MDCT (GE Healthcare, Chicago, Illinois, USA), and a Philips 128-row MDCT scanner (Philips Healthcare, Andover, Massachusetts, USA). MDCT images were acquired using the following parameters: 120kV, 240-260mAs, collimations of 64*0.625, slice thicknesses and increments of 5 mm, and axial reconstruction with 1.25 mm slice thickness and 1 mm slice interval. The patients were kept nil by mouth for 4 hours and later given 600-1000 ml liter of water to drink per-operation scanning. When the followed MDCT performed, patients after curative surgery would be asked for drink water as more as they can. Bowel preparation was not used. MDCT data acquisition of the late atrial and portal venous phase was initiated 10s and 45s after the trigger threshold (100 HU on the abdominal aorta) had been reached. Intravenous non-ionic contrast was administrated (100 ml iopromide 370 mg l/ml; Bayer Schering Pharma ®, Berlin, Germany) with a power injector (Missouri XD 2001, Ulrich GmbH & Co. KG ®, Buchbrunnenweg, Ulm, Germany) at a rate of 2.5 ml/s through an antecubital vein. Image analysis was performed on a workstation with three-dimensional reconstruction software (Advantage Workstation 4.3, GE Healthcare ®, Chicago, Illinois, USA). This allowed the images to be viewed in coronal and sagittal planes, and rotated for comprehensive analysis.

### Histopathology technique and evaluation [31]

Each biopsy sample or radical surgery specimen was fixed in formalin for 24 hours. Hematoxylin and eosin (H&E)-stained slides were then reviewed using a microscope (Olympus BX51 ®, Olympus, Tokyo, Japan) to evaluate the histological type and differentiation of the tumor. Tumor and lymph node status of each subject were defined based on the America Joint Committee on Cancer (AJCC) 7^th^.

### Data collection

The following data were collected: patient demographics [gender, age], preoperative imaging status [ctEMVI, extramural tumor depth (ctEMD), andlymph node status (ctN), and location], postoperative status [tumor status (pT), lymph node status (pN), and tumor differentiation], and synchronous metastases.. Final tumor AJCC stage was determined based on pathology reports and surgery records.

### Preoperative imaging evaluation

Two board-certified radiologists (5 and 3 years of experience in abdominal radiology, respectively), who were blinded to the pathology reports, independently reviewed the scans retrospectively on PACS. Then the two observers reviewed the scans for a third time to reach a consensus for those where disagreement was the final result. All radiologists had been trained to evaluate ctEMVI status and had mastered image reconstruction software allowing them to view images on the coronal and sagittal planes as well as any other planes of interest for a comprehensive analysis.

### Definitions

#### MDCT detected EMVI (ctEMVI)

**Figure 3:**
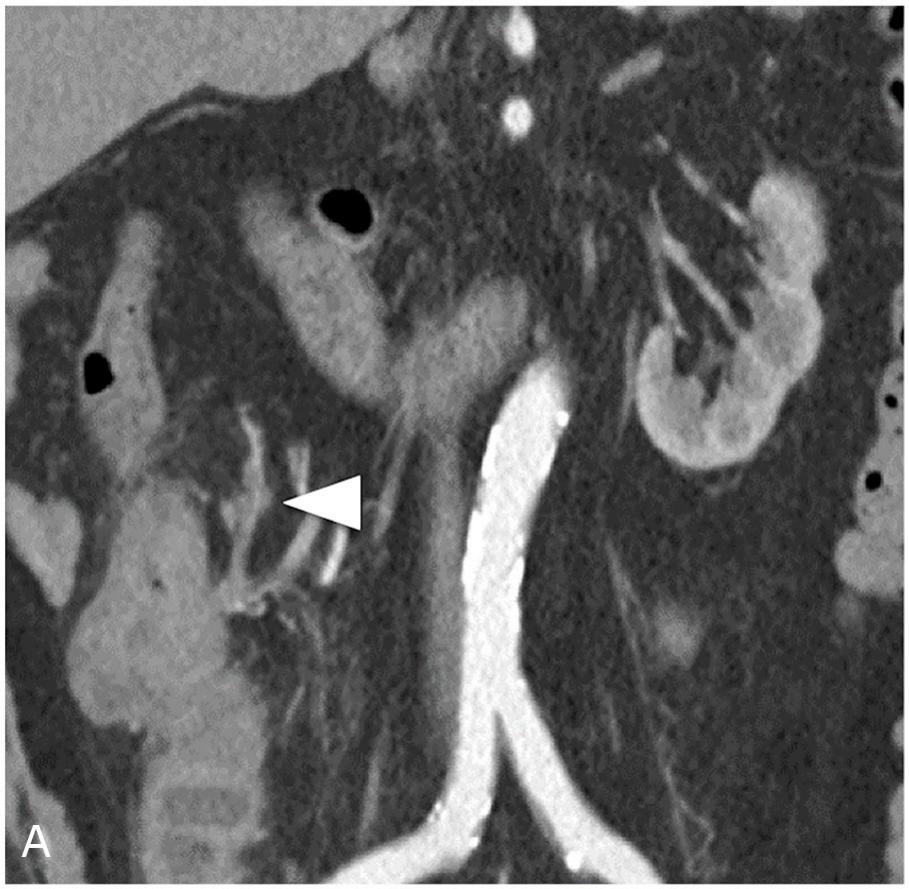

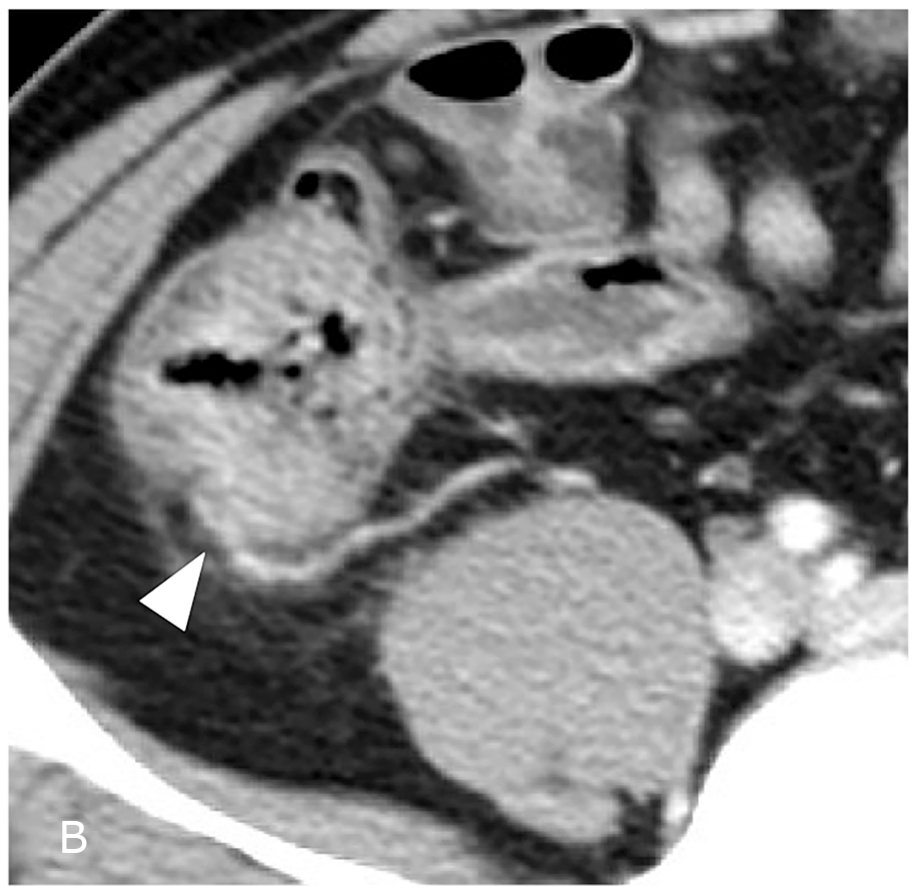
Preoperative MDCT image demonstrates EMVI **(A)** MDCT image shows descending colon cancer of a 73-year-old man. There was tumor density in extramural vessels and the contour and caliber of these vessels are obviously irregular (white arrows). EMVI score 4, Positive. **(B)** MDCT image shows fumigating colon cancer of a 34-year-old man. There were extramural vessels near the tumor, but no tumor density in extramural vessels (white arrows). EMVI score 2, Negative.

Extramural vascular invasion (EMVI) was identified as the presence of malignant cells within blood vessels beyond the muscularis propria [15, 32]. We assigned the criteria of EMVI as a score of 0–3 depending on the radiological features of CT [31]. A score of 0 or 1 corresponded to the absence of ctEMVI, and a score of 2 or 3 corresponded to the presence of ctEMVI. (Table 4, Figure 3).

**Table 4:** ceMDCT classification for EMVI^1^

CT score	CT status	Morphology features on CT	EMVI status
0	Definite No	Absence of tumor extension beyond the colon wall/ Tumor extension through the colon wall but no adjacent vessels (Mesenteric contralateral side)	Negative
1	Suspicious No	Stranding in proximity of vessels but no tumor density in vessels (Mesenteric side)	Negative
2	Suspicious Yes	Similar tumor density in adjacent vessels; vessel expansion by tumor (Mesenteric side)	Positive
3	Definite Yes	Similar tumor density in adjacent vessels; Irregular vessel contour by tumor (Mesenteric side)	Positive

^1^ EMVI: extramural vascular invasion.

#### MDCT detected extramural tumor depth (ctEMD)

ctEMD was defined as the invasion of tumor tissue beyond the tumor margin. ctEMD was documented as positive if the tumor margin was larger than 5 mm, and negative if less than 5 mm.

#### MDCT detected lymph node status (ctN)

MDCT detected positive lymph node was defined as any enhancing nodal mass larger than 1 cm in the short axis, and/or a group of 3 or more nodes, and/or those with irregular borders [8]. Because of location inconformity of lymph nodes between pathology and MDCT scans, we confirmed the positive node patient by patient. This means that if both the pathological report and MDCT scan detected the existence of a positive node, we considered the positive node of pathology to be detected by MDCT.

#### Tumor location

Tumor locations included the ileocecal junction, ascending colon, hepatic flexure, transverse colon, splenic flexure, descending colon, and sigmoid colon. These locations were divided into two groups: right and left. The right group included the ileocecal junction, ascending colon, hepatic flexure, and the right part of the transverse colon. The left group included the splenic flexure, descending colon, sigmoid colon, and the left part of the transverse colon.

#### Pathological tumor status (pT) and lymph node status (pN)

Tumor and lymph node status and stage, all in accordance with the criteria AJCC 7th, were determined based on pathological and surgical records.

#### Tumor differentiation

Histological tumor differentiation classifications consisted of adenocarcinoma and mucinous adenocarcinoma. These classifications were further subdivided into high-medium differentiation and low differentiation.

#### Synchronous metastases (SM)

Based on radiological scans and surgical records, presence of metastases after the initial diagnosis or the development of distant metastases within 3 months after surgery was considered to be synchronous metastases.

### Statistical analyses

Inter-observer agreements regarding the presence or absence of ctEMVI, as well as the ctEMD and ctN, were calculated using Kappa statistical analyses shown as weighted k values. According to Landis and Loch, k values < 0.4 indicate poor agreement; 0.4–0.6, moderate agreement; 0.6–0.8, good agreement; and values > 0.8, indicate excellent agreement [33].

Associations between ctEMVI and other factors such as age, sex, ctN, ctEMD, tumor location, tumor differentiation, pT and pN were analyzed using Chi-squared tests.

Chi-squared and Fisher’s exact tests were used to analyze the associations of synchronous metastases with age, sex, preoperative factors (ctEMVI, ctEMD, ctN and tumor location) and postoperative factors (pT, pN and tumor differentiation). Then, preoperative and postoperative parameters were used to build a multivariable logistic regression model, respectively. Odds ratio (OR) with 95 % confidence interval (CI) was used to express the association of each parameter with metastases. Statistically significant was defined as *P* values less than 0.05 with two-sided.

Data were analyzed using SPSS 22.0 (IBM SPSS, Chicago, Illinois, USA).

## Abbreviations

ceMDCT: contrast-enhanced multiple-row detectors computed tomography
EMVI: extramural vascular invasion
ctEMVI: CT detected extramural vascular invasion
ctN: CT detected lymph node status
ctEMD: CT detected extramural tumor depth
pT: pathological tumor status
pN: pathological lymph node status
SM: synchronous metastases
SD: standard deviation
OR: odds ratio
CI: confidence interval
